# Can self-imposed prevention measures mitigate the COVID-19 epidemic?

**DOI:** 10.1371/journal.pmed.1003240

**Published:** 2020-07-21

**Authors:** Lei Zhang, Yusha Tao, Mingwang Shen, Christopher K. Fairley, Yuming Guo

**Affiliations:** 1 China-Australia Joint Research Center for Infectious Diseases, School of Public Health, Xi’an Jiaotong University Health Science Center, Xi’an, Shaanxi, PR China; 2 Melbourne Sexual Health Centre, Alfred Health, Melbourne, Australia; 3 Central Clinical School, Faculty of Medicine, Nursing and Health Sciences, Monash University, Melbourne, Victoria, Australia; 4 Department of Epidemiology and Biostatistics, College of Public Health, Zhengzhou University, Zhengzhou, Henan, China; 5 Department of Epidemiology and Preventive Medicine, School of Public Health and Preventive Medicine, Monash University, Melbourne, Australia

## Abstract

Yuming Guo and colleagues discuss the research by Teslya et al that highlights the importance of personal preventative measures in avoiding a second wave of the COVID-19 epidemic.

As of 25 May, the severe acute respiratory syndrome coronavirus 2 (SARS-COV-2) pandemic has infected almost 5.2 million individuals, and nearly 340,000 individuals have died of the infection globally. Most governments have implemented strict policies to reduce the movement and social contacts of their populations. The strategies included border closure, reduction in nonessential services, and forbidding crowd gathering to reduce person-to-person contacts. In addition, self-imposed prevention, such as social distancing, handwashing, and face mask usage, are implemented to various extents across affected areas. The findings by Teslya and colleagues in the current issue of *PLOS Medicine* are insightful. The study demonstrates that self-imposed prevention, as a reaction to information dissemination about the coronavirus disease 2019 (COVID-19), can be effective strategies to mitigate and delay the epidemic. In comparison, short-term government-imposed social distancing might only delay the epidemic for the healthcare systems to better prepare for an increasing COVID-19 burden. In an environment where considerable disagreement exists of the optimal way to minimise both mortality from infection and economic harm from control measures, the paper by Teslya and colleagues is most welcome.

Government-initiated intervention strategies can only be relatively short-lived if severe and long-lasting economic and social consequences are to be minimised. Although the 76-day city-wide quarantine in Wuhan, together with weeks of nationwide emergency responses against the epidemic, have largely controlled the SARS-COV-2 in the country, it came with enormous economic consequences that saw its gross domestic product shrunk by 6.8% in the first quarter of 2020 [1]. And unfortunately, the risk of a resurgence of the epidemic from imported cases remains high even with other control measures. To date, as least 6 countries, including China, Germany, Iran, South Korea, Lebanon, and Saudi Arabia, have experienced some resurgence of the epidemic after lifting the lockdown. The work by Teslya and colleagues is particularly relevant to the Chinese experience and suggests that the very successful government-initiated social distancing measures may, at best, delay the epidemic for months [2]. But what Teslya and colleagues’ modelling implies is that with high levels of self-imposed prevention measures, a second epidemic may not occur.

Improving mass awareness is important in facilitating and sustaining these self-imposed interventions [3]. Improving awareness should particularly focus on individuals with a lower awareness such as the elderly, disadvantaged individuals, or those with lower levels of education [4]. The United States data show that ethnic minorities with socioeconomic disadvantages tend to have a higher risk of acquiring COVID-19 [5]. Multiple factors, such as living in population-dense areas, poor personal and environmental hygiene, and low healthcare affordability, contribute to an elevated risk of COVID-19 infection in these populations. Self-awareness of preventive measures is hence more crucial in these populations to prevent a wide-spreading epidemic. Further, governments will need to ensure they reach most of the key groups given that social distancing will have limited some communication. Social media is an effective means of communication to spread COVID-19 epidemic information and improve mass awareness for its prevention, such as promoting proper handwashing. However, the elderly population may not adapt to the rapid development of social media, and conventional means of mass media, such as radio, television, postal mails, and telemarketing, should be used to reach this population. On the other hand, social media can also spread misinformation in what WHO has called an ‘infodemic’, which is marked with excessive false contents, rumours, and disinformation, that may offset the benefits of mass awareness and bring confusion and fear [6].

We agreed that a combination of self-imposed strategies, rather than single isolated prevention, should be advocated to the public. Social distancing and handwashing are effective strategies well accepted by most people, but the importance of face mask usage should not be overlooked. Although there is an ongoing debate on whether it is necessary to wear face masks by nonmedically trained lay individuals, the effectiveness of face masks has been documented in the previous outbreaks of SARS and influenza [7], as well as in clinical settings for COVID-19 [8]. In addition to the prevention of air transmission of aerosol, face masks may also block the frequent contacts between hand and nose. A behavioural study reported that people touch their face 23 times every hour without notice [9]. Further, an accumulating body of modelling studies demonstrating a substantial benefit from the use of a face mask by the public [10–12].

At an international level, there has been an interesting shift in policy recommendation towards face mask usage for the prevention of COVID-19 (Fig 1). In the early stage of the pandemic, China was severely affected and had required all citizens to wear face masks in public spaces in late January. In stark contrast, at the same time, WHO declared there was no evidence that face masks would be effective in protecting healthy individuals [13]. In early April, WHO changed their guidelines, which now indicate that face masks could be effective in limiting the spread of COVID-19 [14]. Other countries are now recommending face cloth coverings to help prevent the transmission of SARS-COV-2. On 15 April, amid the rapidly spreading of the epidemic, New York City declared that face coverings must be used when social distancing is not possible, and Singapore has declared a law that all citizens must wear a mask outside their homes. Based on our estimation, by 4 May, there are approximately 63 countries and territories globally that recommend or require wearing a face mask in public space (Fig 1).

**Fig 1:**
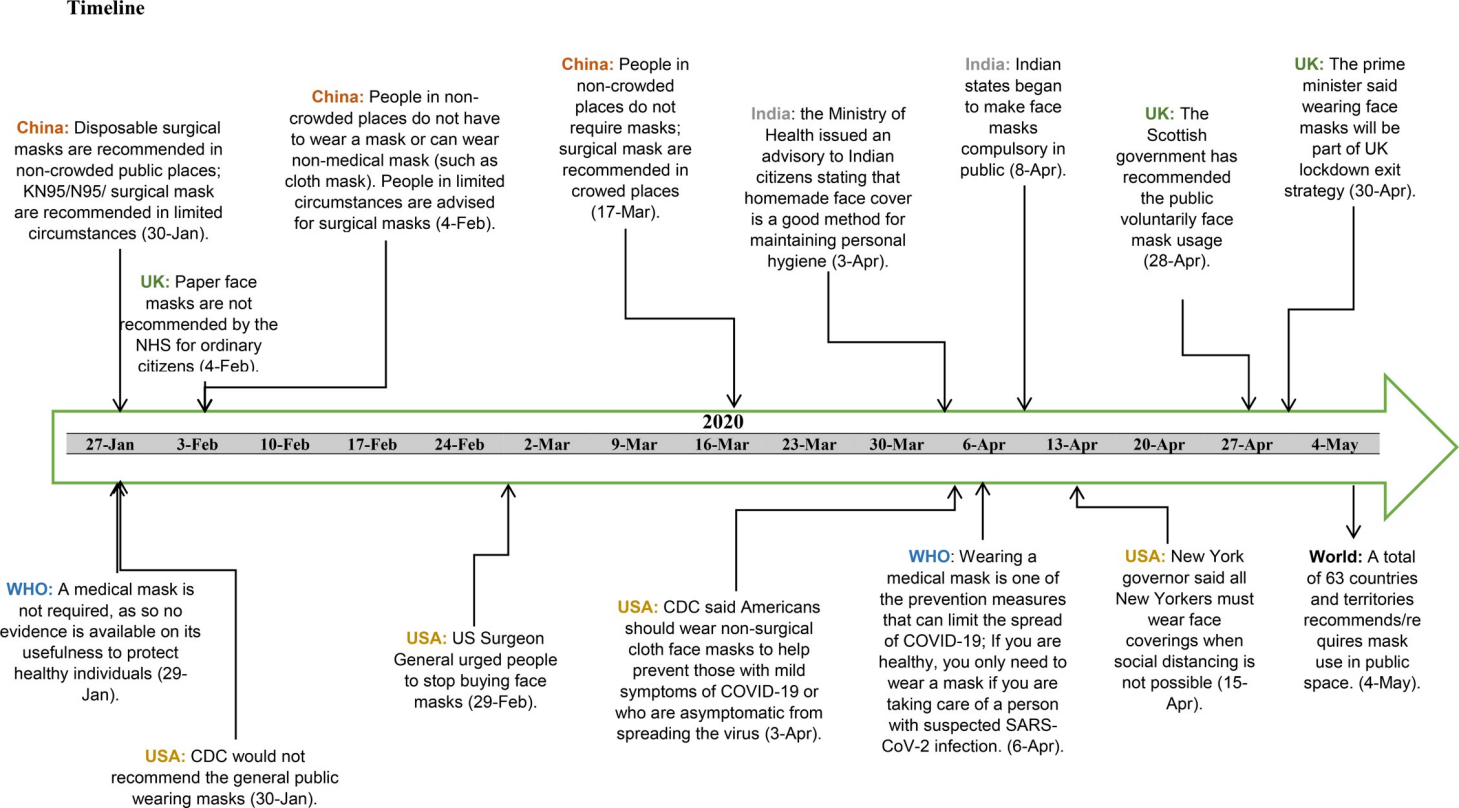
Timeline of policy recommendations for face mask usage by WHO and key countries.

The mass usage of the face mask is not appropriate when the disease burden in the population is as low as it is currently, for example, in Australia and New Zealand. Widespread usage by the general population is likely to lead to shortages and soaring prices that will limit their use by healthcare personnel at high risk [15]. Recommendations about face mask usage in the community should only be made when sufficient supplies are available for healthcare personnel, and there is a rapidly spreading epidemic.

The key messages from the work of Teslya and colleagues are clear. Government-initiated social distancing alone is unlikely to be sufficient for controlling COVID-19 epidemics. It is also important to appreciate that these government programmes, while necessary, come with harsh economic and very substantial social impacts on society. Suicide rates rose by about 5% in men in the year after the global financial crisis [16], and it is likely that the economic impact from COVID-19 will be substantially higher. However, many of the self-imposed prevention strategies have very limited impact on the economy but contribute very significantly to epidemic control and are likely to play a very substantial role in control.

## Abbreviations

COVID-19: Coronavirus Disease 2019
SARS-COV-2: Severe acute respiratory syndrome coronavirus 2

## References

[1] National Bureau of Statistics of China. Preliminary results of gross domestic product (GDP) for the first quarter of 2020. Available from: http://www.stats.gov.cn/tjsj/zxfb/202004/t20200417_1739602.html. [cited 2020 May 10].

[2] Teslya A, Pham TM, Godijk NG, Kretzschmar ME, Bootsma MCJ, Rozhnova G. Impact of self-imposed prevention measures and short-term government-imposed social distancing on mitigating and delaying a COVID-19 epidemic: A modelling study. PLoS Med. 2020;17(7): e1003166 10.1371/journal.pmed.1003166 PMC7373263 32692736

[3] Abdelhafiz AS, Mohammed Z, Ibrahim ME, Ziady HH, Alorabi M, Ayyad M, et al Knowledge, Perceptions, and Attitude of Egyptians Towards the Novel Coronavirus Disease (COVID-19). J Community Health. 2020 4 21 10.1007/s10900-020-00827-7 32318986 PMC7173684

[4] Wolf MS, Serper M, Opsasnick L, O’Conor RM, Curtis LM, Benavente JY, et al Awareness, Attitudes, and Actions Related to COVID-19 Among Adults With Chronic Conditions at the Onset of the US Outbreak: A Cross-sectional Survey. Ann Intern Med. 2020 4 10 10.7326/M20-1239 32271861 PMC7151355

[5] Garg S, Kim L, Whitaker M. Hospitalization Rates and Characteristics of Patients Hospitalized with Laboratory-Confirmed Coronavirus Disease 2019—COVID-NET, 14 States, 3 1–30, 2020 MMWR Morb Mortal Wkly Rep. 2020;69:458–64. 32298251 10.15585/mmwr.mm6915e3 PMC7755063

[6] Limaye RJ, Sauer M, Ali J, Bernstein J, Wahl B, Barnhill A, et al Building trust while influencing online COVID-19 content in the social media world. Lancet Digit Health. 2020 4 21 10.1016/S2589-7500(20)30084-4 32322814 PMC7173823

[7] Offeddu V, Yung CF, Low MSF, Tam CC. Effectiveness of Masks and Respirators Against Respiratory Infections in Healthcare Workers: A Systematic Review and Meta-Analysis. Clin Infect Dis. 2017;65(11):1934–42. 10.1093/cid/cix681 29140516 PMC7108111

[8] Wang X, Pan Z, Cheng Z. Association between 2019-nCoV transmission and N95 respirator use. J Hosp Infect. 2020 3 07 10.1016/j.jhin.2020.02.021 32142885 PMC7134426

[9] Kwok YL, Gralton J, McLaws ML. Face touching: a frequent habit that has implications for hand hygiene. Am J Infect Control. 2015;43(2):112–4. 10.1016/j.ajic.2014.10.015 25637115 PMC7115329

[10] Eikenberry SE, Mancuso M, Iboi E, Phan T, Eikenberry K, Kuang Y, et al To mask or not to mask: Modeling the potential for face mask use by the general public to curtail the COVID-19 pandemic. Infect Dis Model. 2020;5:293–308. 10.1016/j.idm.2020.04.001 32355904 PMC7186508

[11] Zhang L, Shen M, Ma X, Su S, Gong W, Wang J, et al What is required to prevent a second major outbreak of SARS-CoV-2 upon lifting the quarantine of Wuhan city, China. The Innovation. 2020; 1(1): 100006 10.1016/j.xinn.2020.04.006 PMC7237941 33458717

[12] Shen M, Peng Z, Guo Y, Rong L, Li Y, Xiao Y, et al Assessing the Effects of Metropolitan-Wide Quarantine on the Spread of COVID-19 in Public Space and Households. Int J Infect Dis. 2020 5 8 10.1016/j.ijid.2020.05.019. PMC7207105 32416146

[13] World Health Organization, Advice on the use of masks in the community, during home care and in healthcare settings in the context of COVID-19:intermin guidance. Geneva: World Health Organization, 2020 Jan 29. Report No.: WHO/nCov/IPC_Masks/2020.1

[14] World Health Organization, Advice on the use of masks in the context of COVID-19: interim guidance, 6 April 2020. Geneva: World Health Organization, 2020. Report No.: Contract No.: WHO/2019-nCov/IPC_Masks/2020.3.

[15] Feng S, Shen C, Xia N, Song W, Fan M, Cowling BJ. Rational use of face masks in the COVID-19 pandemic. The Lancet Respiratory Medicine. 2020;8(5):434–6. 10.1016/S2213-2600(20)30134-X 32203710 PMC7118603

[16] Chang S-S, Stuckler D, Yip P, Gunnell D. Impact of 2008 global economic crisis on suicide: time trend study in 54 countries. BMJ: British Medical Journal. 2013;347:f5239 10.1136/bmj.f5239 24046155 PMC3776046

